# Conservation of CD44 exon v3 functional elements in mammals

**DOI:** 10.1186/1756-0500-1-57

**Published:** 2008-07-29

**Authors:** Elena Vela, Josep M Hilari, María Delclaux, Hugo Fernández-Bellon, Marcos Isamat

**Affiliations:** 1 Department of R+D+I, Laboratorio Dr. Echevarne, Barcelona, Spain; 2 Zoo-Aquarium de Madrid, Parques Reunidos, Spain; 3 Parc Zoològic de Barcelona, Spain; 4 Fundación Echevarne, Barcelona, Spain

## Abstract

**Background:**

The human CD44 gene contains 10 variable exons (v1 to v10) that can be alternatively spliced to generate hundreds of different CD44 protein isoforms. Human CD44 variable exon v3 inclusion in the final mRNA depends on a multisite bipartite splicing enhancer located within the exon itself, which we have recently described, and provides the protein domain responsible for growth factor binding to CD44.

**Findings:**

We have analyzed the sequence of CD44v3 in 95 mammalian species to report high conservation levels for both its splicing regulatory elements (the 3' splice site and the exonic splicing enhancer), and the functional glycosaminglycan binding site coded by v3. We also report the functional expression of CD44v3 isoforms in peripheral blood cells of different mammalian taxa with both consensus and variant v3 sequences.

**Conclusion:**

CD44v3 mammalian sequences maintain all functional splicing regulatory elements as well as the GAG binding site with the same relative positions and sequence identity previously described during alternative splicing of human CD44. The sequence within the GAG attachment site, which in turn contains the Y motif of the exonic splicing enhancer, is more conserved relative to the rest of exon. Amplification of CD44v3 sequence from mammalian species but not from birds, fish or reptiles, may lead to classify CD44v3 as an exclusive mammalian gene trait.

## Background

**Figure 1:**
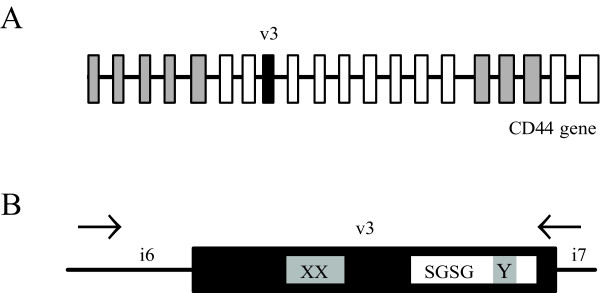
**CD44 exon v3**. A) Genomic structure of human CD44 gene (gray boxes, constitutive exons; white boxes, alternative exons; black box, variable exon v3; black line, introns). B) Schematic representation of exon v3 and its flanking introns (gray boxes, relative location of the XX and Y splicing enhancer motifs; white box, relative location of the nucleotides coding for the GAG binding site and the SGSG motif). Genomic DNA amplification was performed with PCR primers located in intron 6 and in the v3-intron 7 junction (indicated by arrows).

The CD44 family of transmembrane glycoproteins mediates the response of cells to their extracellular microenvironment by regulating growth, survival, differentiation and motility. All human CD44 proteins are encoded by a single, highly conserved gene containing 20 exons, 12 of each undergo alternative splicing [1] (see figure 1A). Complex alternative splicing of the central region of the gene is responsible for the incorporation of the variable domains to shape, predominantly, the extracellular, membrane-proximal stem structure of the protein. The heterogeneity of the CD44 protein products can be further increased by post-translational modifications [1-4]. The sequence encoded in exon v3 contains an optimal Ser-Gly-Ser-Gly (SGSG) consensus motif for modification by heparan sulfate (HS) side chains, to which several heparin-binding proteins attach [5]. This unique HS addition site is critical for CD44v3 isoforms' capacity to bind and present HS-dependent growth factors.

Human variable exon v3 can follow a specific alternative splicing route different from that affecting other variable exons so it can be included in the mRNA together with other variable exons or independently from them [6,7]. This inclusion is regulated by a multisite bipartite exonic splicing enhancer (ESE) consisting in a tandem nonamer (XX motif) and a heptamer (Y motif) that act cooperatively for the efficient recognition of the splice sites [8]. The XX motif is located centrally in the exon while the Y motif is located within the sequence coding for the glycosaminglycan (GAG) binding site immediately downstream from the SGSG motif in v3 (figure 1B).

In order to address the existence and functional nature of the XXY ESE in non-human species we have evaluated the overall level of conservation of CD44 exon v3, including its splicing regulatory elements-the 3' splice site (3'ss), the XXY splicing enhancer- and the GAG binding site, in 95 mammalian species. We also provide data of CD44v3 inclusion into mRNA from peripheral blood samples, by means of RT-PCR, in some representative mammalian taxa with differing levels of conservation of the sequence elements analyzed.

## Methods

Frozen (-80°C) blood samples were selected from the animal tissue bank of the Department of R+D+I, Laboratorio Dr. Echevarne, Barcelona, Spain. Genomic DNA was isolated from 200 μl of blood using the NucleoSpin Blood kit (Macherey-Nagel) following manufacturer's instructions. PCR amplification of CD44v3 was performed with INT6SF and I7wtR primer set or -49v3F and I7wtR primer set (table 1) using PCR Master Mix (Promega). PCR bands of interest were isolated from agarose using the NucleoSpin Extract II kit (Machery-Nagel), sequenced in both directions with the primers used during PCR and the CEQ Dye Terminator Cycle Sequencing Quick Start kit (Beckman Coulter) and analyzed in a CEQ 8800 Genetic Analysis System (Beckman Coulter). All sequences were edited to remove ambiguous base calls and primer sequences and submitted to GenBank.

**Table 1:** Primers

*Primer*	*Region*	*Sequence*
INT6SF	Intron 6	5'-ACCTTCTGTGCCTGATTTTC-3'
I7wtR	Exon v3-intron 7	5'-AATTGATTATTCTTACTGGTGCTGG-3'
-49v3F	Intron 6	5'-GCTTGGCGTCCAGCTCAG-3'
E20F-VI	Exon 20	5'-GGGCAGAAGAAAAAGCTAGTNAT-3'
E20R-QEM	Exon 20	5'-CCAAATGCACCATYTCYTG-3'
13v3F	Exon v3	5'-ATATCATCTCAGCAGGCT-3'
100v3R	Exon v3	5'-TCATCAATGCCTGATCCA-3'

CD44 RT-PCR was performed from total RNA extracted from frozen blood samples with a modification of the QIAamp RNA Blood Mini kit protocol (QIAGEN). Briefly, 150 μl of frozen blood were lysed at 70°C for 10 min with RLT/β-mercaptoethanol buffer containing 4 mg/ml Proteinase K and centrifuged at 10,000 × g for 3 min. 450 μl of the lysate supernatant were mixed with 225 μl of absolute ethanol and loaded in a QIAamp spin column following manufacturer's instructions. Eluted RNA was treated with RQ1 RNase-free DNase (Promega) at 37°C for 30 min and purified following the QIAamp RNA Mini protocol for RNA cleanup (QIAGEN). The first-strand reaction was performed with random primers (Promega) and SuperScript II Reverse Transcriptase (Invitrogen). As control of RNA quality, total CD44 isoforms were amplified with degenerate E20F-VI and E20R-QEM primer set (table 1) using GC-Rich PCR System (Roche).

In order to amplify CD44v3 containing isoforms, PCR primers were designed based on a multiple sequence alignment containing the sequences corresponding to the 95 mammalian species. Exon v3 positions that showed full conservation were identified and selected to locate the 3' ends of the primers ensuring perfect matches. According to this, v3 amplification was perfomed with primers 13v3F and 100v3R (table 1) and PCR Master Mix (Promega). As control of complete genomic DNA digestion, non reverse-transcribed RNAs were tested amplification negative with primers 13v3F and 100v3R.

Molecular conservation analyses were conducted using the MEGA version 3.1 software [9] and sequence logos were generated with the WebLogo application [10].

## Results

### CD44v3 sequencing

There is little sequence data available for CD44 variable exons from most animal species. The orthologue prediction for human CD44 in Ensembl *release 48 *provides v3 exon sequence for 16 species of mammals. In order to increase the data available we studied CD44 exon v3 in most of the animal samples stored in our tissue bank.

A region that enabled amplification of CD44v3, was located by a Blast search against multiple species using a human genomic fragment spanning intron6-v3-intron7. Alignment of sequences corresponding to the 10 nt at the 3' end of the INT6SF primer matched perfectly in *Macaca mulatta*, *Canis familiaris*, *Oryctolagus cuniculus *and *Pan troglodytes *and presented a single nucleotide missmatch in *Mus musculus*, *Rattus norvegicus*, *Loxodonta africana *and *Bos taurus*. Likewise, primer I7wtR matched perfectly with the exception of *Mus musculus *where there was a single nucleotide missmatch. We tested primers INT6SF and I7wtR in *Loxodonta africana*, *Canis familiaris*, *Bos taurus*, *Oryctolagus cuniculus *and in non-mammalian species such as *Spheniscus humboldti*, *Psittacus erithacus *and *Varanus niloticus*. Sequence confirmation of PCR products showed that only mammalian species amplified CD44v3. We failed to amplify v3 (exonic and/or flanking intronic sequences) from *Spheniscus humboldti*, *Psittacus erithacus*, *Cygnus atratus*, *Threskiornis aethiopicus*, *Dacelo novaguineae*, *Ciconia ciconia*, *Amazona aestiva*, *Guaruba guarouba*, *Varanus niloticus*, *Sparus aurata*, *Merluccious merlucius *and *Plesionika edwarsii*. In the absence of v3 specific PCR amplification we cannot evaluate the presence/absence of v3 nor the lack of conservation of primer regions. Lack of v3 amplification from bird, fish or reptile DNA is in agreement with the absence of exon v3 from available genomes of *Gallus gallus *[GenBank: NC_006092], *Xenopus tropicalis *[Ensembl: ENSXETG00000007556] and *Gasterosteus aculeatus *[Ensembl: ENSGACG00000011430].

In view of this, our sample set has been restricted to 95 mammalian species distributed in 29 families. The region amplified comprises, relative to the known human CD44v3 sequence, a 5' partial fragment of intron 6 and a 3' partial fragment of exon v3 (117 nucleotides out of 126) (see figure 1B). The resulting sequences are shown in Additional file 1.

### CD44v3 splicing regulatory elements conservation

**Figure 2:**
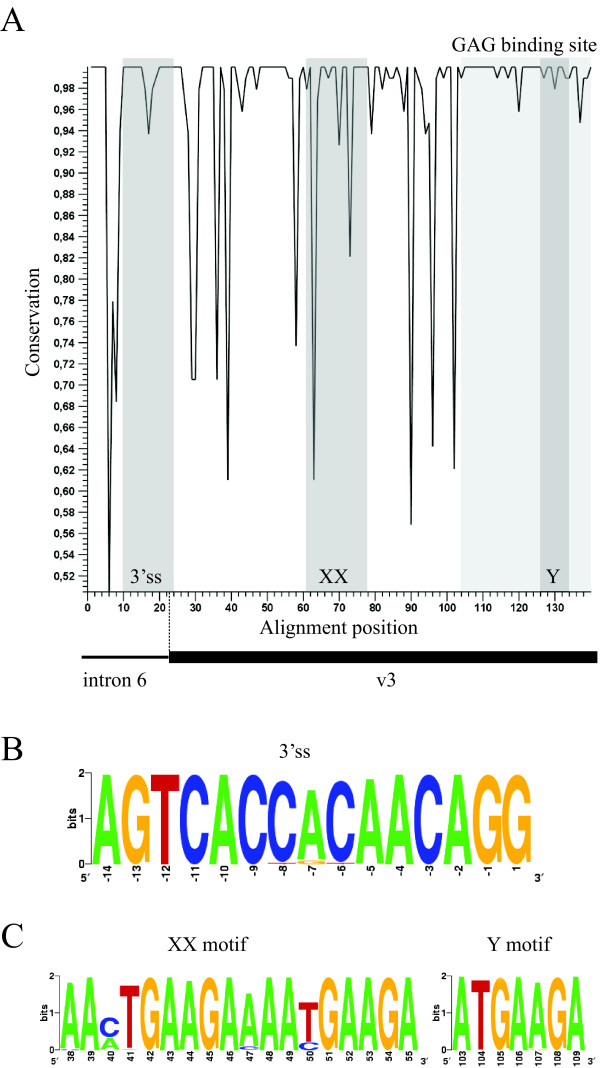
**Nucleotide sequence conservation**. A) Top, line plot displaying the level of conservation at each position of the alignment of the 95 species analyzed (dark gray boxes, location of 3'ss, XX and Y splicing enhancer motifs; light gray box, GAG binding site). Bottom, schematic representation of the genomic region aligned. B) Sequence logo of the 3'ss. Positions are numbered according to the exon sequence. C) Sequence logo of the XX and Y splicing enhancer motifs. Positions are numbered according to the exon sequence.

We have compared sequences of 140 nucleotides long spanning 23 nucleotides of intron 6 and 117 nucleotides of exon v3, in the mammalian species listed in Additional file 1. The genomic region amplified enables the analysis of the CD44v3 splicing regulatory elements, namely, the 3'ss and the XXY ESE [8]. The level of conservation at each position of the alignment in the 95 species analyzed is shown in figure 2A. Sequence alignment of exon v3 in these species reveals 69 (59%) conserved and 48 (41%) variable residues. In this way, the 95 species studied are represented by 28 different nucleotide sequences.

The 3'ss is fully conserved in 84 out of 95 species. The rest of species (11 out of 95) have single-nucleotide substitutions at positions -5 (n = 1), -6 (n = 2), -7 (n = 6) or -8 (n = 2) (see figure 2B). The functional significance of these varying positions in the splice site is addressed below by means of v3 expression analysis in peripheral blood.

The percentage of conserved residues in the ESE and in exon v3 is 68% and 59%, respectively. These values suggest that the level of conservation of the ESE with respect to the exon is of the same range (figure 2A). Upon ESE dissection, XX reveals higher variability relative to Y (figure 2A and 2C) which remains conserved in all species with the exception of *Elephas maximus*, *Loxodonta africana *and *Ursus arctos *which present synonymous variations (see Additional file 1). If we consider the XXY ESE as a whole unit of 25 nucleotides long, such unit is represented by 13 different sequences whose relative frequency is shown in table 2. The most common sequence is *# *10, present in 42% of the species tested and followed by *# *2, present in 28% of the species. The latter corresponds to the previously described human sequence [8] and it is also detected in other primate families. The third most common sequence is *# *8, present in 11% of the species. The analysis of the XXY sequences reveals 17 (68%) conserved and 8 (32%) variable residues of which most are single-nucleotide substitutions. Within the XX motif, positions 3 (conserved in 58 sequences out of 95), 4 (92 out of 95) and 10 (88 out of 95) are represented by 3 different nucleotides. The positions that have been functionally shown elsewhere to decrease CD44v3 inclusion in mutant expression constructs (X mutant: AAATGggtA and Y mutant: ATGggtA) [8] remain invariant, corroborating their functional importance during splicing.

**Table 2:** Sequences representing the XXY ESE in CD44v3

*XXY ID*	*Sequence (XX_Y)*	*N Species*	*freq (%)*	N changes*
1	AAaTGAAGAAAAcGAAGA_ATGAAGA	2	2.11	2
2	AAaTGAAGAAAATGAAGA_ATGAAGA	27	28.42	1
3	AAaTGAAGAAAATGAAGA_ATGAgGA	2	2.11	2
4	AAaTGAAGAcAATGAAGA_ATGAAGA	3	3.16	2
5	AAaTGAgGAAAATGAAGA_ATGAAGA	1	1.05	2
6	AACaGAAGAAAAcGAAGA_ATGAAGA	2	2.11	2
7	AACcGAAGAAAAcGAAGA_ATGAAGA	1	1.05	2
8	AACTGAAGAAAAcGAAGA_ATGAAGA	11	11.58	1
9	AACTGAAGAAAATGAAGA_AcGAAGA	1	1.05	1
10	AACTGAAGAAAATGAAGA_ATGAAGA	40	42.11	0
11	AACTGAAGAcAATGAAGA_ATGAAGA	3	3.16	1
12	cAgTGAAGAAAATGAAGA_ATGAAGA	1	1.05	2
13	cAgTGAAGAtAAcGAAGA_ATGAAGA	1	1.05	4

Each XXY sequence is represented by an identification number (XXY ID). Variable positions relative to most common sequence (#10) are indicated in lower case. Number of species representing each sequence and its relative frequency are displayed.

*Total number of changes relative to #10.

### CD44v3 GAG binding site conservation

**Figure 3:**
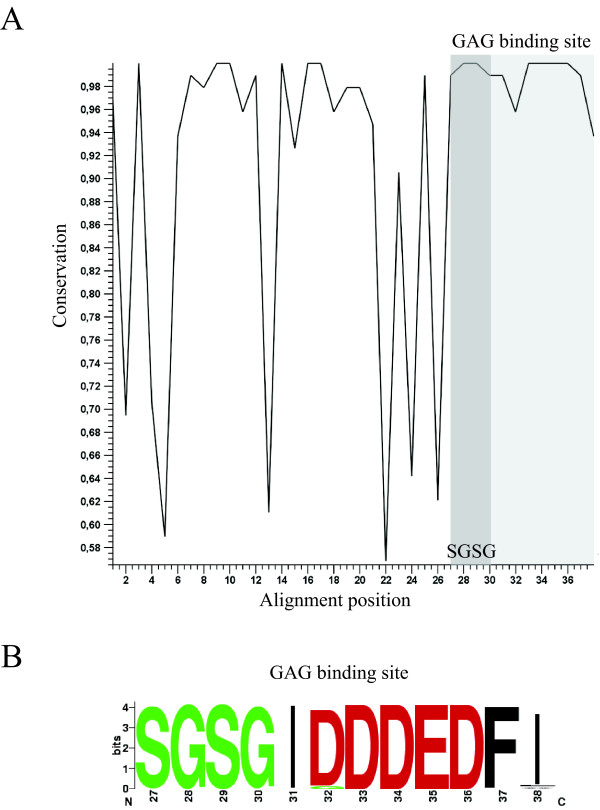
**Amino acid sequence conservation**. A) Line plot displaying the level of conservation at each position of the alignment of the 95 species analyzed (dark gray box, location of SGSG motif; light gray box, GAG binding site). B) Sequence logo of the GAG binding site. Positions are numbered according to the v3 coded domain.

Considering v3's reading frame (codon start = 3), the 117 nucleotides of CD44 exon v3 translate into a 38 amino acid extracellular protein domain. Amino acid sequence alignment of this domain from our data set shows 12 (32%) conserved and 26 (68%) variable residues classifying the 95 species studied into 28 different amino acid sequence groups. This alignment also reveals full conservation of the SGSG motif (figure 3A) with the exception of *Tursiops truncatus *and *Oryctolagus cuniculus *where the sequences have been changed to SGSD and PGSG, respectively (see Additional file 1). Bourdon et al [11] identified the amino acid sequence homology around the SG dipeptide sites that serves as GAG attachment sites in the core proteins of proteoglycans. The core protein must contain acidic amino acids on the amino-terminal side of the sequence SGXG, where X stands for any amino acid. The S is the most critical of the invariant residues and the relative importance of the residues is S > first G > second G > acidic residues. According to this, only the rabbit's v3 domain would not be expected to bind HS.

Human exon v3 contains acidic residues both upstream and downstream of the SGSG motif. The eight amino acids located downstream of the SGSG site consist of acidic residues flanked by hydrophobic residues that are necessary for the specific addition of HS at this site [12]. The species analyzed maintain a conserved GAG binding site both at the nucleotide (see figure 2A) and the amino acid level (figure 3B) implying that the secondary and/or tertiary structure around the SGSG motif is critical to initiate HS attachment, and this may have further contributed to the conservation of the Y ESE motif contained therein in all species tested.

### CD44v3 expression in mammalian species

CD44v3 has been reported to be constitutively expressed in human peripheral blood cells, irrespective of their activation status [13-15]. We have used peripheral blood accordingly as a model to evaluate the expression of CD44v3 isoforms in some taxon-representative mammalian species. The RT-PCR results (see Additional files 1 and 2) show that there is no correlation between sequence variation within the 3'ss or the ESE and lack of v3 expression in peripheral blood. All species tested have revealed v3 expression implying that the conservation observed is sufficient to maintain v3 inclusion. The human CD44 protein contains an unique HS binding site coded by CD44 exon v3 [16], therefore enabling only CD44v3 containing isoforms to carry HS side chains and to bind and present heparin-binding growth factors and cytokines. On this basis, presence of a conserved GAG attachment site in all mammals studied may reflect a similar function [17,18] for CD44v3 in these species.

In addition to mammals, CD44 constitutive exons are also found in birds, amphibians and fish as described in public databases although their expression in certain tissues in such taxa has not been addressed. In conclusion, we have obtained CD44v3 sequence from 95 mammalian species but have failed to amplify the homologous fragment from bird, reptile or fish species, in agreement with the lack of CD44 variable exons from available genome sequences of model organisms in these taxa. This implies that CD44v3 appears to be an exclusive mammalian gene trait. The sequence conservation observed in our dataset would support a common origin and function for this exon in all mammals. Furthermore, CD44v3 sequence conservation in mammalian species enables maintenance of functional splicing regulatory elements and the GAG binding site. The level of conservation of the sequence encoding the GAG binding site, which in turn contains the Y motif of the ESE analysed, is higher than the overall level found for the rest of the exon. Whether this phenomenon is due to purifying selection pressure contributed by the GAG attachment domain alone or in conjunction with the Y motif of the ESE remains undetermined. Functional inclusion of CD44v3 has also been demonstrated in peripheral blood from mammalian species representative of the different sequence variations observed, implying *in vivo *use of exon v3 in these species. Further work is required to search for the exact evolutionary origin of CD44 exon v3 in mammals.

## Abbreviations

SGSG, Ser-Gly-Ser-Gly; HS, heparan sulfate; ESE, exonic splicing enhancer; GAG, glycosaminglycan; 3'ss, 3' splice site.

## Competing interests

The authors declare that they have no competing interests.

## Authors' contributions

EV conceived and designed the study, carried out the molecular genetic studies and sequence analysis and was the principal author of the manuscript. JMH participated in the design of the study, completed analyses, interpreted findings and reviewed the manuscript. MD and HFB participated in taxonomically certified sample acquisition and reviewed the manuscript. MI participated in the design and coordination of the study, interpreted findings and revised critically the manuscript for its final approval.

## Supplementary Material

Additional file 1 Summarized results. GenBank accession number, taxonomic information, nucleotide sequence, GAG binding site motif and v3 expression results are listed for each sample. Nucleotide sequence is split into exon v3, intron 6, 3' splice site and XXY ESE. The XXY identification number (XXY ID) and its relative representation among species (XXY freq) are indicated. Results of v3 expression are displayed as positive expression (++) or data not available (n.a.). Click here for file

Additional file 2 Raw RT-PCR expression data of CD44v3 containing isoforms. CD44v3 containing isoforms were amplified from equivalent amounts of cDNA (RT+) and non-retrotranscribed RNA (RT-) digested with DNase. All samples were amplified in the same experiment with primers 13v3F and 100v3R. Negative control (C-) consisted of amplification in the absence of template cDNA/RNA. Positive control (*H. sapiens*) consisted of a human sample where v3 is reported to be expressed in normal peripheral blood cells [13-15]. PCR product size (bp) is compared to molecular weight ladder (MW). Interpretation of results: samples were considered positive for CD44v3 expression when the net intensity of the amplification of the RT+ was higher than the RT-counterpart. Amplification in some RT-samples is compatible with residual genomic DNA. Click here for file

## References

[B1] Screaton GR, Bell MV, Jackson DG, Cornelis FB, Gerth U, Bell JI (1992). Genomic structure of DNA encoding the lymphocyte homing receptor CD44 reveals at least 12 alternatively spliced exons. Proc Natl Acad Sci U S A.

[B2] Stamenkovic I, Amiot M, Pesando JM, Seed B (1989). A lymphocyte molecule implicated in lymph node homing is a member of the cartilage link protein family. Cell.

[B3] Bajorath J (2000). Molecular organization, structural features, and ligand binding characteristics of CD44, a highly variable cell surface glycoprotein with multiple functions. Proteins.

[B4] Ponta H, Sherman L, Herrlich PA (2003). CD44: from adhesion molecules to signalling regulators. Nat Rev Mol Cell Biol.

[B5] Bennett KL, Jackson DG, Simon JC, Tanczos E, Peach R, Modrell B, Stamenkovic I, Plowman G, Aruffo A (1995). CD44 isoforms containing exon V3 are responsible for the presentation of heparin-binding growth factor. J Cell Biol.

[B6] Roca X, Mate JL, Ariza A, Munoz-Marmol AM, von Uexkull-Guldeband C, Pellicer I, Navas-Palacios JJ, Isamat M (1998). CD44 isoform expression follows two alternative splicing pathways in breast tissue. Am J Pathol.

[B7] Vela E, Roca X, Isamat M (2006). Identification of novel splice variants of the human CD44 gene. Biochem Biophys Res Commun.

[B8] Vela E, Hilari JM, Roca X, Munoz-Marmol AM, Ariza A, Isamat M (2007). Multisite and bidirectional exonic splicing enhancer in CD44 alternative exon v3. Rna.

[B9] Kumar S, Tamura K, Nei M (2004). MEGA3: Integrated software for Molecular Evolutionary Genetics Analysis and sequence alignment. Brief Bioinform.

[B10] Crooks GE, Hon G, Chandonia JM, Brenner SE (2004). WebLogo: a sequence logo generator. Genome Res.

[B11] Bourdon MA, Krusius T, Campbell S, Schwartz NB, Ruoslahti E (1987). Identification and synthesis of a recognition signal for the attachment of glycosaminoglycans to proteins. Proc Natl Acad Sci U S A.

[B12] Greenfield B, Wang WC, Marquardt H, Piepkorn M, Wolff EA, Aruffo A, Bennett KL (1999). Characterization of the heparan sulfate and chondroitin sulfate assembly sites in CD44. J Biol Chem.

[B13] Forster-Horvath C, Bocsi J, Raso E, Orban TI, Olah E, Timar J, Ladanyi A (2001). Constitutive intracellular expression and activation-induced cell surface up-regulation of CD44v3 in human T lymphocytes. Eur J Immunol.

[B14] Bendall LJ, Bradstock KF, Gottlieb DJ (2000). Expression of CD44 variant exons in acute myeloid leukemia is more common and more complex than that observed in normal blood, bone marrow or CD34+ cells. Leukemia.

[B15] Legras S, Gunthert U, Stauder R, Curt F, Oliferenko S, Kluin-Nelemans HC, Marie JP, Proctor S, Jasmin C, Smadja-Joffe F (1998). A strong expression of CD44-6v correlates with shorter survival of patients with acute myeloid leukemia. Blood.

[B16] Jackson DG, Bell JI, Dickinson R, Timans J, Shields J, Whittle N (1995). Proteoglycan forms of the lymphocyte homing receptor CD44 are alternatively spliced variants containing the v3 exon. J Cell Biol.

[B17] van der Voort R, Taher TE, Wielenga VJ, Spaargaren M, Prevo R, Smit L, David G, Hartmann G, Gherardi E, Pals ST (1999). Heparan sulfate-modified CD44 promotes hepatocyte growth factor/scatter factor-induced signal transduction through the receptor tyrosine kinase c-Met. J Biol Chem.

[B18] Sherman L, Wainwright D, Ponta H, Herrlich P (1998). A splice variant of CD44 expressed in the apical ectodermal ridge presents fibroblast growth factors to limb mesenchyme and is required for limb outgrowth. Genes Dev.

