# Supplementary figures and images for: Conservation of CD44 exon v3 functional elements in mammals
Source: BMC Res Notes. 2008 Jul 29;1:57. doi: 10.1186/1756-0500-1-57 (PMC2531186; doi:10.1186/1756-0500-1-57)

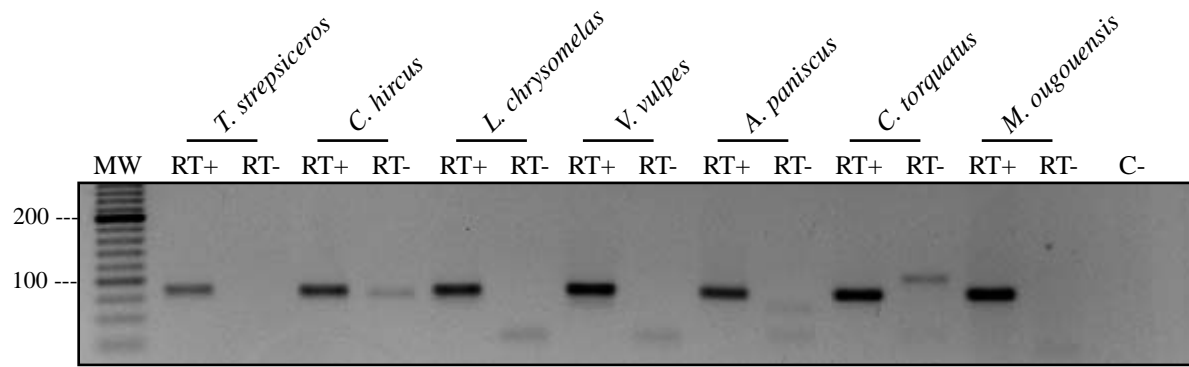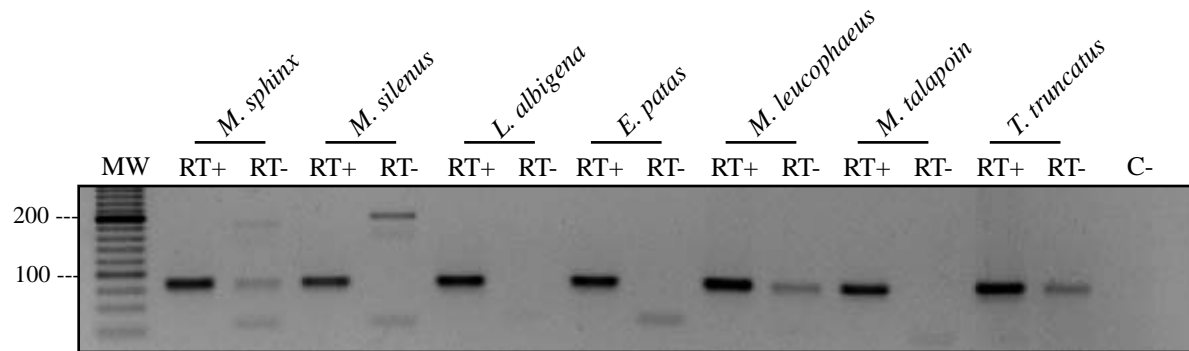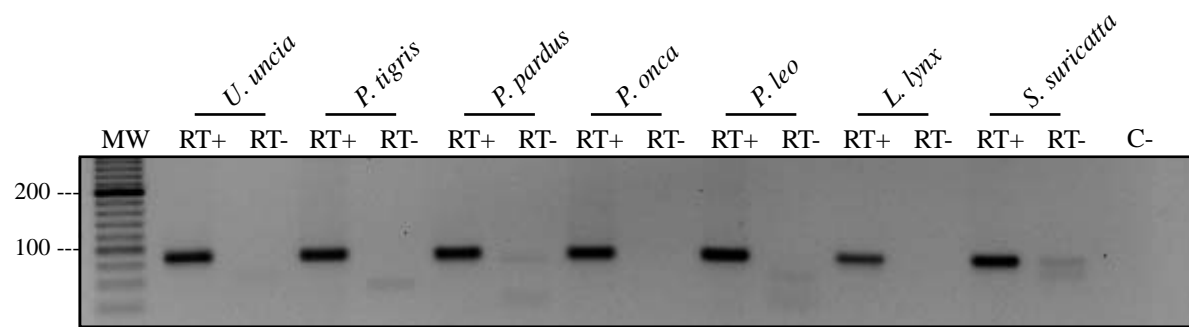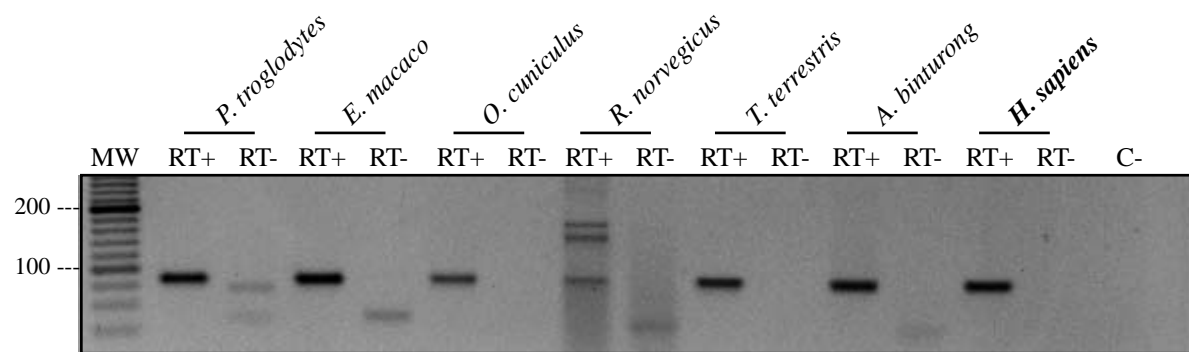

Supplement: Additional file 2 — Raw RT-PCR expression data of CD44v3 containing isoforms. CD44v3 containing isoforms were amplified from equivalent amounts of cDNA (RT+) and non-retrotranscribed RNA (RT-) digested with DNase. All samples were amplified in the same experiment with primers 13v3F and 100v3R. Negative control (C-) consisted of amplification in the absence of template cDNA/RNA. Positive control (H. sapiens) consisted of a human sample where v3 is reported to be expressed in normal peripheral blood cells [13-15]. PCR product size (bp) is compared to molecular weight ladder (MW). Interpretation of results: samples were considered positive for CD44v3 expression when the net intensity of the amplification of the RT+ was higher than the RT-counterpart. Amplification in some RT-samples is compatible with residual genomic DNA. [file 1756-0500-1-57-S2.pdf]
